# Presentation and Repair of Serial Misdiagnosed Congenital Patellar Dislocation

**DOI:** 10.7759/cureus.20082

**Published:** 2021-12-01

**Authors:** Hiep Nguyen, Elizabeth Mekler, Ann Truong, Nghi Nguyen, Enjolina Iqbal, Ariel Kidron

**Affiliations:** 1 Emergency Medicine, Nova Southeastern University Dr. Kiran C. Patel College of Osteopathic Medicine, Fort Lauderdale, USA; 2 Orthopedic Surgery, Nova Southeastern University Dr. Kiran C. Patel College of Osteopathic Medicine, Fort Lauderdale, USA; 3 Medicine, Nova Southeastern University Dr. Kiran C. Patel College of Osteopathic Medicine, Fort Lauderdale, USA; 4 Orthopedic Surgery, Xuyen A Hospital, Ho Chi Minh, VNM

**Keywords:** musculoskeletal rehabilitation, general surgery, congenital birth defect, patella dislocation, ortho surgery

## Abstract

Congenital patellar dislocation (CPD) is a rare deformity in children that involves a laterally displaced patella. While potentially identified in early childhood using diagnostic imaging techniques, it is often misdiagnosed at birth, creating pain and mobility issues as the child grows. Dislocation of the patella is permanent and manually irreducible, often manifesting with flexion contracture of the knee, genu valgum, external tibial torsion, and foot deformity. Surgical correction is the treatment of choice in order to prevent future sequelae. We herein present a case of CPD in a four-year-old Asian male who was initially misdiagnosed before undergoing successful surgical repair using the Roux-Goldthwait technique.

## Introduction

Congenital patellar dislocation (CPD) is a highly rare condition, presenting as a manually irreducible lateral patellar bone dislocation with a flexion contracture and valgus tibial deviation [1]. It can simultaneously occur alongside other disorders such as Ellis-van Creveld syndrome, Down syndrome, and Rubinstein-Taybi syndrome [2]. The condition may go undiagnosed early on since the dislocated patella will not appear on standard X-rays until after the bone ossifies. Thus, clinicians must possess a high index of suspicion for dislocation in order to make the correct diagnosis. CPD must be resolved surgically and may cause significant physical disability if uncorrected [3]. Based on existing literature, this skeletal deformity was hypothesized to potentially result from improper anterior rotation of the quadricep during fetal myotome development [4].

In this paper, we present an individual case of a young male presenting with CPD after many misdiagnoses. He was eventually treated successfully via using the Roux-Goldthwait surgical technique, which is a very common and efficient surgical method with minimal postoperative complications. Based on a report of prior surgical treatments, all cases showed no signs of recurrence and significant effectiveness in improving knee function [5]. Thus, we specifically emphasize this precise methodology and procedure of the technique in this paper in order to further understand the mechanisms of its success for CPD.

## Case presentation

**Figure 1:**
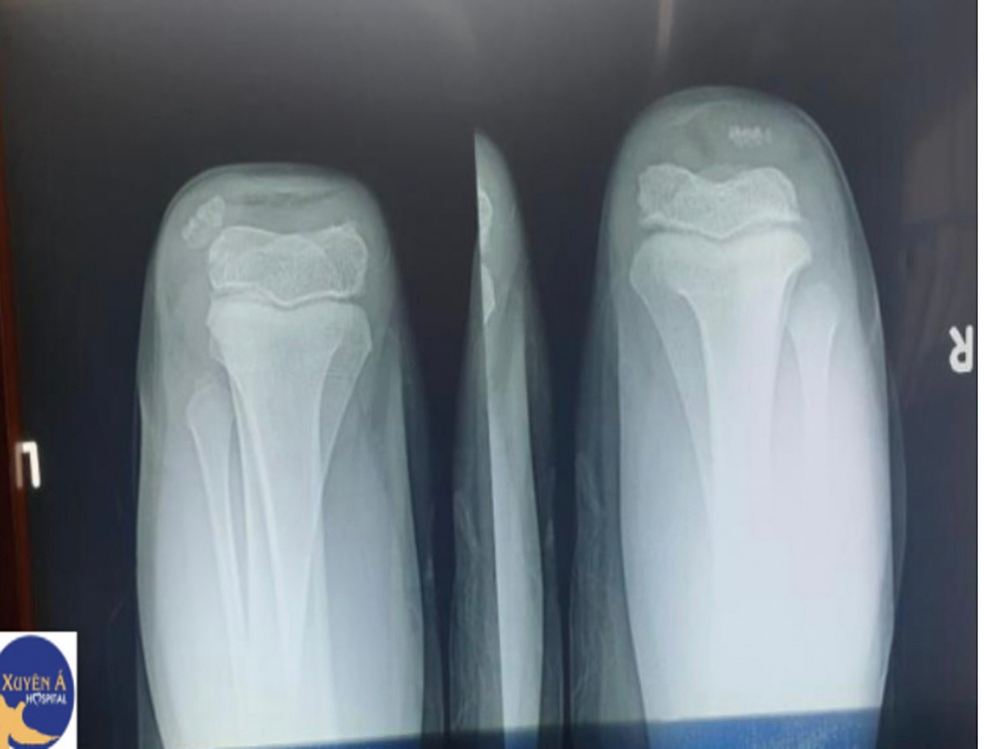
X-ray reveals dislocated patella on the left knee

We present a four-year-old Vietnamese male with Down syndrome for chronic pain and inflammation of the left knee for one year. Per the patient's medical record, he was previously diagnosed with juvenile arthritis at multiple hospitals and clinics, for which he had received several pharmacotherapies without symptom relief. Medications included acetaminophen, ibuprofen, corticosteroid, and methotrexate. As the result of persistent, non-resolving pain in the left knee, the patient was then referred to Xuyen A Hospital for further investigation. Physical examination revealed epiphyseal dysplasia, funnel chest, contracture elbow joint, and diastrophic dysplasia. The left knee displayed genu valgum and flexion contracture. Besides the chronic pain, the patient reported no limit in the left knee’s range of motion. X-ray of both knees was performed, yielding the diagnosis of CPD (Figure 1).

After an adequate risk-to-benefit assessment, a surgical intervention appeared to be most appropriate, and the Roux-Goldthwait technique was employed. In this distal realignment procedure, the patellar tendon was split vertically. The lateral half of the patellar tendon was then moved inferomedially to the medial half to form a “Roux” pattern. Finally, the lateral half was attached to the tibia. This pulls the dislocated patella over to the center, thus simultaneously preventing excess lateral shift. This technique is similar to other well-known patellofemoral procedures, such as Fulkerson, Maquet, and Trillat, without disrupting open physes in immature muscle-skeletal structures [3]. In addition, the surgical team also performed the lateral release procedure, V-Y lengthening of the quadriceps, and posterior release of the knee. These minor adjustments aimed to relieve the patient’s pain and discomfort postoperatively. Morphine, ibuprofen, and topical mupirocin were prescribed to minimize pain and wound infection. The patient was discharged a week later with splinting of the knee. Physical therapy was scheduled once a week, and the caregivers were instructed on wound care and dressing. 

**Figure 2:**
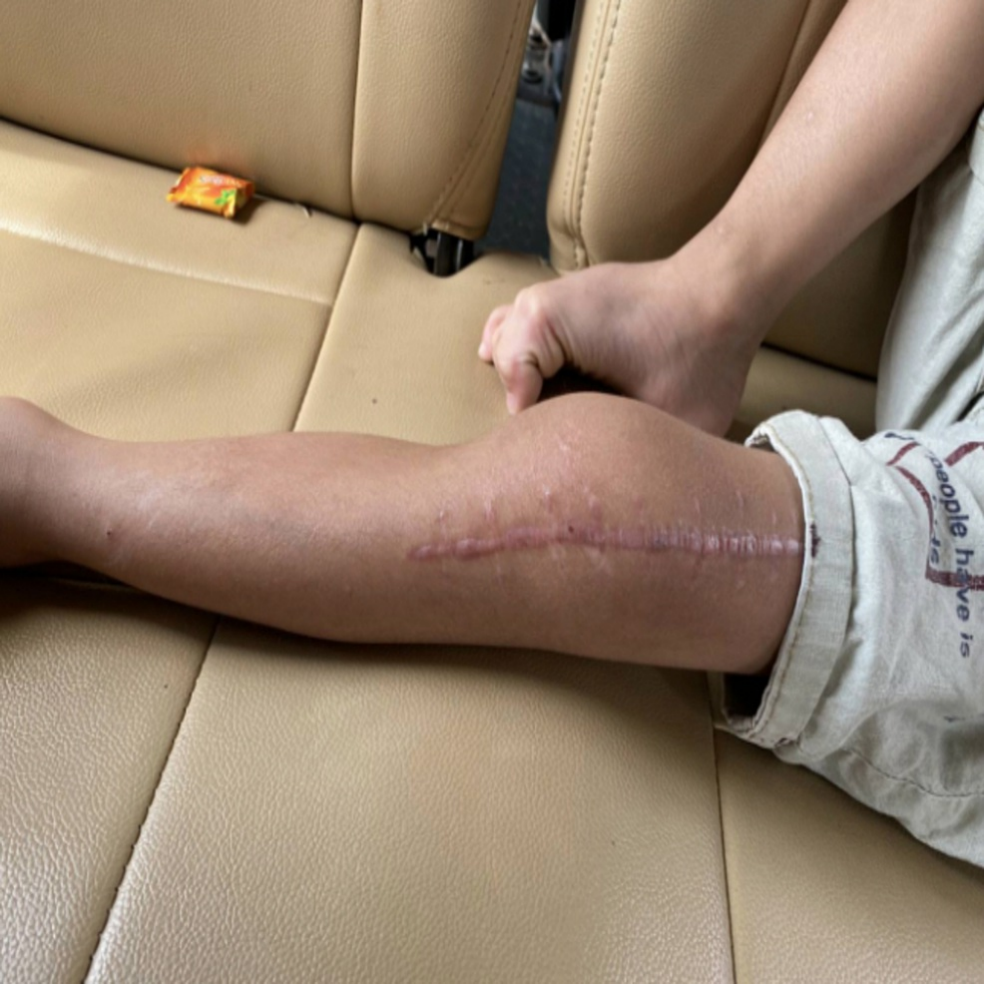
Healed scar of the patient three months after surgery

At three months follow-up, the incision appeared to heal well without any infection or rupture. A hypertrophic scar was formed along the incision line, which is a normal reaction of damaged skin from surgery cuts (Figure 2). The patient started to walk in small steps without assistance. As part of the recovery process, the patient was refrained from fast-paced and weight-bearing activities, such as running or lifting heavy objects, for approximately up to 12 months in order to heal and recover properly.

## Discussion

Congenital dislocation of the patella, also known as fixed lateral dislocation of the patella, is a very rare deformity. The condition presents at birth with lateral dislocation of the patella that cannot be reduced manually. CPD can have multiple clinical presentations such as habitual dislocation of the patella or juvenile arthritis. CPD typically presents with a flexion contracture at the knee, genu valgum, foot deformity, and external tibial torsion. Although this disorder can be diagnosed at birth, it tends to be misdiagnosed due to low suspicion of the disorder, lack of patellar visibility on plain radiographs, and difficulty in palpating the hypoplastic patella in the lateral position [4]. CPD is associated with arthrogryposis, chondrodysplasia punctata, dyschondrosteosis, chondro-osteodystrophy, thrombocytopenia-absent radius syndrome, fibular hemimelia, Larsen syndrome, and Williams-Beuren syndrome [6].

When presenting at birth, the dislocation is often misdiagnosed as a neuromuscular disease or muscular dystrophy. It is unknown what the true statistics of CPD are since the confirmed cases are sporadic. Approximately, the incidence is estimated as 1/100,000, with a higher prevalence in girls than boys [7]. Historically, clinicians and researchers find it difficult to calculate the true prevalence of CPD. This is due to the fact that the condition has many overlapping symptoms with other diseases such as Down syndrome, Rubinstein-Taybi syndrome, and Ellis-Van Creveld syndrome. Moreover, CPD is also considered a symptom of the aforementioned syndrome, adding more challenge to the diagnosis of the disorder in clinical settings [8].

This case report presents a Vietnamese male with Down syndrome, who was originally misdiagnosed with juvenile arthritis at one year old. The Roux-Goldthwait technique was implemented for correcting the patella’s location. The benefit of this technique is that it allows the surgeon to adjust the position of the patella according to each presented case in order to improve tracking. This technique is similar to other well-known patellofemoral procedures, such as Fulkerson, Maquet, and Trillat, without disrupting the open physes in immature muscle-skeletal structures [4].

In addition, lateral release, V-Y lengthening of the quadriceps, and posterior release of the knee were also performed on the patient during the surgery to relieve any pain and discomfort that may result from the surgery. During the lateral release procedure, a group of lateral tendons is cut in order for the kneecap to move into the proper groove. This helps relieve the patient of pain and pressure from any tightening in the lateral retinaculum tissue on the outside of the kneecap [3]. The V-Y lengthening of the quadriceps involves freeing both the medial and lateral aspects of the extensor mechanism from the quadriceps tendon and its extensive attachment to the underlying femur. To accomplish this, the medial and lateral retinacula are divided. The iliotibial band (ITB) should also be released or lengthened if the tibia is in valgus and external rotation. After sharp dissection of the lateralis and medialis, the quadriceps tendon is cut in a V-shaped manner. The lateral retinaculum is freed from attachments to the femur. With the knee flexed to approximately 60° of flexion, the quadriceps tendon is repaired in its lengthened position, giving the appearance of a V to Y proximal progression. Finally, the medialis and lateralis are reattached to the lengthened quadriceps tendon [9]. 

As with most osteopathic diseases, early diagnosis and intervention are critical. The most common treatment is surgery. Though surgery is the ultimate proper treatment for CPD, other non-invasive treatments in the early stages of the disease include casts, braces, and physiotherapy in order to improve flexural contracture [2]. However, surgical intervention is the preferred treatment of choice in order to avoid late sequelae and prevent early degenerative changes in the knee.

## Conclusions

In such a rare condition as CPD, diagnosis can be delayed due to low suspicion and invisible patella on X-rays early in life. Flexion contracture of the knee, genu valgum, external tibial torsion, and foot deformity in pediatric patients should raise the clinician’s suspicion of CPD. Once the diagnosis is confirmed, an early surgical operation is an effective treatment for relieving pain and allowing children to continue development. Surgical treatment before one year of age allows for the reconstruction of almost normal knee function and provides the patient with a normal gait without delay at the onset of walking. Neglected cases require more extensive excisions of contractures to ensure reduction of the patellar dislocation and more complicated surgical reconstructions to ensure patellar stability. Early detection will improve the prognosis of the disorder and enhance patients’ quality of life. In this case report, the patient recovered successfully from CPD and benefited from combined surgical techniques and postoperative physical therapy after suffering from multiple misdiagnoses for multiple years.
